# Regulation of germline stem cell proliferation downstream of nutrient sensing

**DOI:** 10.1186/1747-1028-1-29

**Published:** 2006-12-06

**Authors:** Patrick Narbonne, Richard Roy

**Affiliations:** 1 McGill University, Department of Biology, 1205 Dr. Penfield Ave, Montréal, Québec, H3A 1B1, Canada

## Abstract

Stem cells have recently attracted significant attention largely due to their potential therapeutic properties, but also because of their role in tumorigenesis and their resemblance, in many aspects, to cancerous cells. Understanding how stem cells are regulated, namely with respect to the control of their proliferation and differentiation within a functional organism, is thus primordial to safely profit from their therapeutic benefits. Here, we review recent advances in the understanding of germline stem cell proliferation control by factors that respond to the nutritional status and/or insulin signaling, through studies performed in *C. elegans *and *Drosophila*. Together, these data uncover some shared fundamental features that underlie the central control of cellular proliferation within a target stem cell population in an organism. These features may indeed be conserved in higher organisms and may apply to various other stem cell populations.

## Background

The development of multi-cellular organisms requires a continual source of differentiating cells to populate fields within diverse tissues. Similarly, following tissue damage, the replacement of lost cells often relies on the proliferation and subsequent differentiation of a population of pluripotent "stem" cells set aside from other cells within this tissue. In order to maintain their population, stem cells must self-renew at each division, which can be accomplished through asymmetric division to generate two different daughter cells – one that resembles the mother (a stem cell), and one that is committed to another differentiated fate. Alternatively the division can result in the formation of two identical daughter cells that are indistinguishable from the mother. This symmetric mode of division enables stem cells to increase in numbers during development, or following an injury [1].

Stem cells occupy a specific microenvironment which is referred to as the niche, wherein they receive the extrinsic signals required to maintain their undifferentiated identity. These signals differ among the various stem cell types, but their role in maintaining the stem cell population is critical, and their expression defines the boundaries of the niche [2].

The study of the regulation of the *C. elegans, Drosophila*, and mouse Germline Stem Cell (GSC) populations during development and adulthood has revealed a number of important molecular mechanisms that govern the interactions between stem cells and their niche [3]. Briefly, a short-range signal(s) generated by the niche cell(s) – the Distal Tip Cell (DTC) in *C. elegans*, the cap and hub cells in the *Drosophila *ovary and testis, respectively, and the Sertoli cells in the mouse testis – prevents nearby GSCs from differentiating. In fact, these extrinsic cues activate a molecular cascade within the GSCs that targets the activity of specific transcription factors and/or translational regulators, which in turn alter gene expression to specify and maintain GSC identity.

Under optimal growth conditions, GSCs divide continuously throughout development and adulthood, initially to increase in numbers and later to provide a constant supply of differentiating germ cells. Under these optimal circumstances, the rate at which GSCs divide appears to be primarily dependent on intrinsic factors and on their interaction with the niche cell(s). In fact, signaling from the niche cell(s) not only physically determines the size of the GSC population, but also affects the rate at which GSCs proliferate, depending on the level at which it regulates GSC identity [4-6]. The limiting intrinsic factors are very poorly defined, but recent advances suggest that the timing of stem cell division may be regulated by a microRNA-dependent down regulation of Dacapo, a p21/p27 Cyclin-Dependent Kinase (CDK) inhibitor, thereby relaxing controls on the G_1_/S transition [7]. That is, *Drosophila *GSCs lacking *dicer-1 *(*dcr-1*) function, the loss of which completely impairs microRNA processing, are delayed at the G_1_/S boundary, and this delay is dependent on Dacapo [8,9].

When environmental conditions are unfavorable to growth however, the rate at which organisms develop is delayed, owing to a general slowing in cell growth and division. This likely occurs as a result of a direct lack of critical nutrient resources required for macromolecular synthesis, but also through nutrient sensing and the active inhibition of energy consuming pathways, such as those involved in cell growth and division, presumably to conserve limiting resources. Several intracellular and intercellular molecular cascades play a role in this active response to adverse growth conditions, including the insulin, AMPK, and TOR signaling pathways [10-13]. It is therefore likely that the GSCs of starved animals follow similar rules as the soma, and their growth/division rate may thus be delayed under such conditions.

The GSCs contain the information that will be transmitted from generation to generation, therefore their genetic integrity is critical and must be guarded from deleterious mutations. The precious treasure that they store is thus subject to additional protective measures that are not utilized in somatic cells. Consistent with this, it is now widely accepted that transposon silencing mechanisms operate much more efficiently in the germ line compared to the soma to prevent deleterious effects caused by aberrant insertion and/or expression of sequences derived from these elements [14]. In addition to transposition events however, many other sub-optimal circumstances may increase mutational susceptibility, including nutrient deprivation [15]. It is therefore expected that under these conditions, GSCs become quiescent in order to minimize the risk of acquiring deleterious mutations due to driving cell division during periods of insufficient energy or resources to appropriately complete the cell cycle.

## Nutrient stress blocks stem cell divisions

In most organisms examined to date, GSC divisions are delayed when nutritional resources become limiting. For example, sterols are essential for growth [16], but *C. elegans *cannot synthesize them *de novo*, and instead must metabolize exogenous sterols to meet this requirement. When cholesterol levels are insufficient, the brood size of *C. elegans *is markedly reduced due to a defect in germline proliferation and differentiation [17]. This correlates with a study that clearly demonstrated that female *Drosophila *GSCs and their progeny uniformly adjust their proliferation rates in response to nutrition, such that no particular developmental stage accumulates in the germarium of poorly fed animals [18]. Therefore, GSCs must sense nutrient quality and/or abundance, or alternatively they must be capable of reading the general metabolic status of the organism, to adjust their division rate accordingly.

## Insulin signaling regulates the rate of GSC divisions

**Figure 1:**
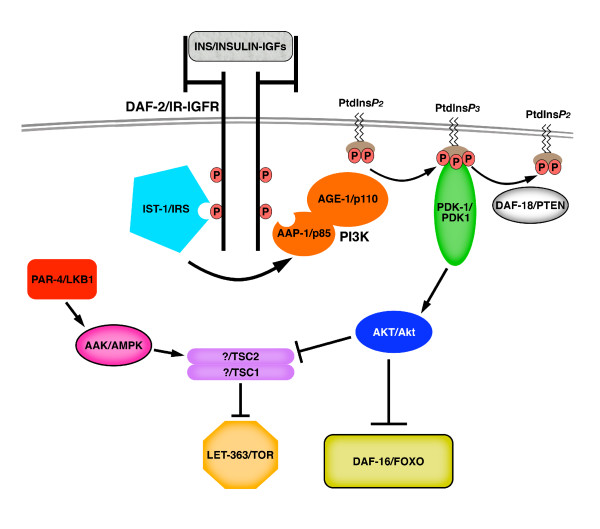
**Linking the insulin-like, AMPK and TOR signaling pathways upstream of GSC quiescence**. Upon insulin-like receptor activation, PtdInsP3 kinase (PI3K) phosphorylates PtdIns*P*_*2*_. This activity is counteracted by PtdInsP3 phosphatase (DAF-18/PTEN). PtdIns*P*_*3 *_activates, in a PDK-1/PDK1-dependent manner AKT/Akt, which phosphorylates and thereby prevents the nuclear translocation of the DAF-16/FOXO transcription factor. In *Drosophila *and mammals, Akt and AMPK act antagonistically to regulate TOR signaling through inhibitory and activating phosphorylation of TSC2, respectively. Arrows indicate activation; bars inhibition. Based on [11, 59, 85, 87, 88].

The general metabolic status of multi-cellular organisms is monitored predominantly by insulin-like signaling [13]. In *C. elegans*, encountering poor environmental conditions during early post-embryonic life, including limited nutritional resources and high population density, triggers the entry into an alternative developmentally-suspended stage called dauer, which is specialized for long-term survival and dispersal. To understand how this developmental switch is regulated, large-scale screens have been carried out to isolate mutants that constitutively enter dauer, or that are unable to execute this developmental switch [19,20]. Several highly conserved components of insulin signaling have been identified from these initial screens. That is, disrupting the function of genes encoding positive components of the *C. elegans *insulin-like cascade, such as the insulin-like growth factor (IGF) receptor ortholog (*daf-2*) [21], the catalytic subunit of PtdIns3-kinase (*age-1*) [22], the PtdIns*P*3-dependent kinase (*pdk-1*) [23], or Akt/PKB (*akt-1/2*) [24] results in a down regulation of the metabolic rate and induces constitutive dauer arrest. In contrast, altering the function of components that act antagonistically to the insulin-like signaling cascade, including PtdIns3-phosphatase PTEN (*daf-18*) [25], or the FOXO-like forkhead transcription factor (*daf-16*) [26,27] disrupts the ability of animals to enter dauer (Figure 1).

In animals in which the level of insulin-like signaling is severely reduced, GSC divisions progressively slow down during preparation for dauer [28], similar to what occurs in somatic tissues, to finally completely arrest such that no cell divisions occur during the dauer diapause. Similarly, reducing insulin-like signaling later in life, following the window of competence to execute dauer development has been bypassed, also inhibits gamete production quite dramatically [29]. Therefore, the level of insulin-like signaling somehow impinges, directly or indirectly, on the rate at which GSCs divide.

In *Drosophila*, insulin-like signaling also drastically impinges on GSC division rate. In fact, while a homolog of the vertebrate Insulin Receptor Substrate (IRS) 1–4, CHICO, is required for female fertility [30], reduced activity of the *Drosophila insulin receptor *(*dinr*) specifically in the GSCs strongly attenuates their rate of division [31]. Thus, the degree of activation of the insulin-like signaling pathway, like the nutritional status, regulates the division rate of GSCs. Furthermore, GSCs require *dinr *and *chico *to properly adjust their division rate with nutrient availability [18,31]. Therefore, nutrient depletion must impinge on GSC divisions by reducing insulin-like signaling within the GSCs, thereby inhibiting their proliferation under these sub-optimal growth conditions.

## Nutrients regulate GSC division rate through an insulin-dependent neuro-endocrine signal

In mammals, the insulin receptor is activated following its association with an insulin molecule secreted by pancreatic β-cells in response to high blood sugar in order to inhibit hepatic glucose production, while also stimulating glucose uptake in muscles and adipose tissues [13]. The insulin and insulin receptor superfamilies comprise several members, including the insulin-like growth factors (IGFs) and their receptors (IGFRs) among others, all of which carry diverse functions [32]. In lower organisms such as *Drosophila *and *C. elegans *however, there are several insulin-like peptides that are mainly expressed in neurons and either positively or negatively regulate the activity of a unique insulin/IGF-1 receptor homolog [33,34].

In *Drosophila*, there are seven *insulin-like peptide *(*dilp1-7*) genes, all of which activate the single IGFR homolog (*dinr*) and thereby promote growth [33,35]. In adult females, DILPs are mainly expressed in two clusters of neurosecretory cells in the brain [36], and the expression of some of these is modulated by nutrient availability [35]. Either the ablation of these DILP-producing cells or the prevention of DILP secretion through impairment of the *Drosophila α-endosulfine *(*dendos*) gene reproduce the delay in GSC proliferation caused by the removal of the *dinr *gene specifically from the germ line [31,37]. Together, these results suggest that the nutritional status regulates DILP expression in a collection of discrete head neurons, and that these DILPs are secreted, transported, and bind directly to DINR on the surface of the GSCs to control their division rate in the *Drosophila *female.

In *C. elegans*, the pathway works somewhat differently. There are 38 predicted insulin-like peptides that are also predominantly expressed in the nervous system, some of which antagonize and some of which activate the unique insulin-like receptor homolog [34,38,39]. Experiments designed to restore the function of the insulin-like receptor (*daf-2*), PtdIns3-kinase (*age-1*), or of the downstream target of the pathway, a FOXO-like forkhead transcription factor (*daf-16*), in specific tissues have demonstrated that *daf-2 *and *age-1 *activity in neurons is sufficient to sustain reproductive development in *daf-2 *or *age-1 *mutant animals, respectively [40-42]. Furthermore, restoring *daf-16 *function specifically in the neurons *of daf-16; daf-2 *double mutants, is sufficient to induce dauer development and concomitantly block GSC divisions [[43], our unpublished data]. It remains unclear, however, whether neuronal *daf-16 *activity is similarly sufficient to couple GSC proliferation with reduced insulin-like signaling levels in a post-dauer situation. Furthermore, the nature of this insulin-dependent neuro-endocrine signal that would stimulate GSC divisions remains elusive. Part of a reasonable hypothesis may be that elevated neuronal insulin-like signaling levels influence the production of a sterol-derived hormone by the cytochrome P450 DAF-9 in the hypodermis and/or in a pair of neuroendocrine cells. This hormone may in turn affect a nuclear hormone receptor called DAF-12, thereby promoting reproductive development [44-47]. However, other factors must be implicated since *daf-2; daf-12*(0) double mutants, despite their inability to execute dauer and completely block GSC divisions under reduced insulin-like signaling, grow into sterile adults [46,48,49], indicating that insulin-like signaling levels regulate GSC divisions and germline development, at least in part, through a *daf-12 *independent mechanism.

## Insulin levels do not seem to affect niche-GSC signaling

A puzzling question remains whether this insulin-like-regulated signal also affects the manner with which the niche communicates with the GSCs. In *C. elegans *and *Drosophila *the niche is considered to actively promote proliferation of the GSCs, while also inhibiting their differentiation [4-6,50]. But during sub-optimal growth conditions it would seem counterintuitive that the niche signal(s) would continue to stimulate proliferation of the GSCs while a second signal would be required to inhibit them from dividing in response to these environmental cues. In *C. elegans*, a Delta/Serrate-like ligand called LAG-2, expressed by the niche cell (DTC), activates a Notch receptor (GLP-1) in the GSCs, thereby promoting their proliferation, as opposed to their differentiation [50]. This cascade does not seem to be affected by changes in insulin-like signaling however, since both the ligand and the receptor continue to be expressed when insulin-like signaling is reduced and GSCs become quiescent during dauer development [28]. However, it is possible to reconcile these observations if the niche signal(s) in fact does not directly promote proliferation, but rather specifies GSC identity, the fate of which is more prone to proliferation. It seems logical that GSC identity must be maintained even when these cells are not actively dividing.

## PtdIns3-phosphatase PTEN regulates GSC divisions in a FOXO-independent manner

The PtdIns3-phosphatase PTEN acts downstream of the insulin-like receptor in every organism examined thus far, counteracting the activity of PtdIns3-kinase. Loss of PTEN activity, therefore, results in increased PtdIns*P*_*3 *_levels, and these increased levels are sufficient to completely suppress all the phenotypes of insulin-like receptor mutants in *C. elegans *and *Drosophila *[51,52]. These results suggest that the effects observed due to variations in the activity of the insulin-like receptor are mediated through its influence on the abundance of PtdIns*P*_*3*_. Elevated PtdIns*P*_*3 *_levels activate a complex that includes Akt/PKB in a PDK1-dependent manner, which in turn phosphorylates and thereby inhibits a FOXO forkhead transcription factor from entering the nucleus (Figure 1) [53]. Although experiments performed in mammalian cells identified other Akt/PKB phosphorylation targets, including TSC2 in the mTOR growth pathway [10,54,55], until very recently it was believed that the activity of the *C. elegans *FOXO homolog *daf-16 *fully mediated the effects of reduced insulin-like receptor (*daf-2*) activity, again because its removal completely suppresses the effects of *daf-2 *mutations on the development of this multi-cellular organism [27,39]. However compelling evidence now demonstrates that some *daf-18*/PTEN-dependent; *daf-16*/FOXO-independent regulation of GSC divisions occurs in *C. elegans*.

*C. elegans *hatchlings do not begin post-embryonic development and their two initial GSCs do not proliferate until the animals start feeding [56]. This quiescence of the GSCs in starved L1 larvae requires the activity of *daf-18*/PTEN, and the inappropriate divisions that occur in *daf-18 *mutants are suppressed by mutations in *age-1*/PtdIns3-kinase or *akt-1*/Akt/PKB. In contrast, a *daf-16*/FOXO null mutation does not bypass the food requirement for GSC proliferation in starved L1 larvae [57]. Similarly, while *daf-18*/PTEN is required in all circumstances to appropriately down regulate the proliferation of the GSCs during dauer development, *daf-16*/FOXO is almost fully dispensable under certain conditions [28]. Moreover, both the inhibition of the proliferation of normal GSCs in growing larvae and of the tumorous GSCs in adult *gld-1 *mutants provoked by reduced insulin-like receptor activity is not completely suppressed by *daf-16*/FOXO null mutations [28,58]. Together, these results suggest that reduced activity of the insulin-like receptor negatively regulates the rate at which GSCs divide, at least in part, through FOXO-independent PTEN and/or Akt/PKB targets.

## Mysterious G_2_/M arrest of low insulin-induced quiescent GSCs

In mammalian cell culture, insulin signaling affects the cell cycle machinery largely by regulating Akt/PKB activity, which is required for progression through both G_1_/S and G_2_/M checkpoints [59]. As previously mentioned, the timing of adult stem cell divisions appears to be mediated by two G_1_/S regulators: p21 and p27 [7], while a large part of the Akt/PKB-dependent G_1_/S regulation is believed to occur through the regulation of these two CDK inhibitors [59]. It seems plausible therefore that GSC quiescence in organisms under nutrient stress occurs at the G_1_/S checkpoint through the inhibition of Akt/PKB-dependent p21/p27 down regulation. Consistent with this, RNAi depletion of a *C. elegans *p21/p27 homolog (*cki-1*) induces GSC hyperproliferation during dauer formation [60], but not during early reproductive development, when nutrients are not limiting [61], implicating it in GSC division control specifically during unfavorable growth conditions. However, observations suggest that GSCs do not arrest in G_1_, but rather at the G_2_/M checkpoint in starved/insulin-like compromised *C. elegans *larvae. Namely, the quiescent GSCs of dauer larvae have twice the DNA content of g_1 _arrested somatic cells [28]. Also, even when both p21/p27 CDK inhibitor homologs (*cki-1 *and *cki-2*) are depleted by RNAi in starved L1 larvae, their GSCs do not divide [62]. Moreover, the GSCs of starved L1 animals have replicated DNA content with condensed chromosomes and duplicated centrosomes; centrosome duplication being specific to S-phase [57]. Together, these data indicate that the insulin-regulated quiescence of the GSCs occurs at the G_2_/M checkpoint, at least in *C. elegans*, although G_1_-specific CDK inhibitors may contribute to the deceleration of GSC divisions associated with environmental stress.

It has been suggested that this surprising result may reflect differences in the mechanisms of cell cycle regulation when the cellular response is coordinated at the organismal level, as opposed to within individual cells [63]. The relevance or significance of this developmentally regulated G_2_/M-arrest of GSCs in response to nutrient depletion however remains unclear. Interestingly, mammalian Embryonic Stem (ES) cells lack a G_1_checkpoint and instead accumulate in S and G_2 _phases after irradiation, at least in part, as a result of compromised Chk2 (a central G_1 _checkpoint mediator) function. This difference between somatic and ES cells has been proposed to contribute to their reduced mutational frequencies, perhaps through favoring the apoptosis of mutant cells over their arrest and repair [64,65]. Consistent with this, a large proportion of ES cells undergo apoptosis after treatment with antimetabolite or genotoxic agents [64]. It is therefore possible that the G_2 _arrest of GSCs in starved animals favors the apoptotic elimination of those cells that have accumulated mutations during the insult over their repair, thereby preventing their transmission to the next generation.

## The LKB1/AMPK cascade links GSC division rate with insulin levels

PTEN is an important tumor suppressor that is among the most commonly mutated genes in most types of human cancer. Also, germline PTEN mutations result in related, dominantly inherited, cancer predisposing syndromes [66]. The downstream targets of the insulin-regulated cascade that couples GSC proliferation with nutritional status are therefore of great interest, potentially representing novel mediators of PTEN signaling that contribute to its tumor suppressive properties.

A forward genetic approach in *C. elegans *identified *aak-2 *as a downstream effector that links GSC proliferation rate with insulin-like signaling levels [28]. The screening strategy took advantage of the developmentally-regulated establishment of the complete GSC quiescence associated with dauer development, such that dauer animals arrest with a characteristic gonad size. Like in *daf-18*/PTEN mutants, the germ line of *aak-2 *mutant dauers is hyperplasic. *aak-2 *encodes a homolog of the α2 catalytic subunit of a heterotrimeric complex called AMP-activated protein Kinase (AMPK) in humans. AMPK is best characterized as an intracellular "metabolic master switch" that turns OFF energy consuming pathways and turns ON alternative energy producing pathways in response to an increase in the AMP:ATP ratio, to restore energy balance [12]. RNAi depletion of the other catalytic (α1) AMPK subunit (*aak-1*) gives a phenotype that is similar to *aak-2*(RNAi), while the inactivation of both subunits results in significantly more pronounced germline hyperplasia, indicating an additive function of the two catalytic AMPK subunits [28].

In addition to being activated allosterically by AMP, AMPK requires at least one key activating phosphorylation at a very conserved site to become fully catalytically active [12]. The major AMPK-activating kinase was identified as LKB1/STK11 [67], a tumor suppressor that causes cancer predisposition in humans [68,69]. As one might predict, the inactivation of the *C. elegans *LKB1 homolog (*par-4*) causes germline hyperplasia in dauer, with a severity similar to that of *aak-1; aak-2 *double mutants. Interestingly, the requirement for *aak-2 *is cell autonomous, suggesting that the LKB1-AMPK cascade functions within the GSCs to regulate proliferation, likely in response to the neuro-endocrine signal downstream of insulin-like signaling [28]. This observation reveals the significance of this LKB1-dependent AMPK phosphorylation in a developing animal, and suggests that the requirement for this very highly conserved LKB1-AMPK cascade in insulin-dependent regulation of GSC division rate may function in other organisms, including humans.

Intensive biochemical studies, most of which were performed in cultured cells, have identified a molecular cascade that links both Akt/PKB and AMPK to the regulation of the mTOR growth pathway. Briefly, Akt/PKB and AMPK antagonistically regulate the activity of a TSC1-TSC2 complex, another human tumor suppressor [70], through direct phosphorylation of TSC2 [55,71-73], such that when insulin signaling is elevated and the AMP:ATP ratio is low, the TSC complex is antagonized by Akt/PKB, and is not activated by AMPK. In turn, the TOR pathway is activated, thereby promoting protein synthesis and cell growth (Figure 1) [11,74]. Interestingly, mutations in the *C. elegans *TOR ortholog (*let-363*) cause larvae to arrest with an underdeveloped germ line, although the animals do not resemble dauer larvae [75]. Furthermore, TOR signaling is required for vitellogenesis and egg development in response to nutritional signals resulting from blood ingestion in female mosquitoes [76]. Given that the rate at which vitellogenesis proceeds is tightly coupled to that of GSC proliferation and DILP signaling in *Drosophila *[31,35], it is likely that TOR signaling couples GSC division rate with nutrient status in insects. To date, however, no direct evidence suggests the involvement of TOR signaling in insulin-dependent regulation of GSC division rate, although based on the biochemical and cellular interactions described above, it is tempting to speculate that the insulin-Akt/PKB and LKB1-AMPK cascades together target TOR activity to adjust the rate of GSC divisions according to the nutritional status of the organism.

The mechanisms at work in unicellular models and tissue culture do not always reflect the complexity associated with development typical of multi-cellular organisms, and it will become a major challenge to account for all the details that remain unanswered, particularly in *C. elegans*. First, as previously mentioned, unlike in *Drosophila*, insulin-like receptor activity is not required within the worm germ line to sustain the robust GSC proliferation associated with reproductive development [41,42]. This suggests that neural insulin-like peptides do not directly control the rate of GSC divisions in this organism and imply a second, yet uncharacterized, neuro-endocrine signal. Furthermore, the phenotypical and molecular links between insulin-like and TOR signaling still lack experimental support in this organism. That is, no obvious TSC1/2 ortholog has been clearly identified in *C. elegans*, while there are clear phenotypic differences between the effects of mutations in these different pathways on development, despite several commonalities, including growth arrest [74,75]. Perhaps the most puzzling finding is the G_2 _arrest of quiescent GSCs, which does not fit with the widely accepted view that nutrient depletion and TOR signaling affect G_1_/S progression. That is, in yeast and mammalian cells, treatment with rapamycin (an inhibitor of TOR signaling) induces G_1 _arrest [77-81]. It shall therefore be a priority to determine whether this G_2 _arrest is an exception or a rule in GSC regulation through a more detailed examination of higher organisms, and whether TOR signaling could somehow participate in this process. Finally, genetic evidence suggests the involvement of additional, yet unidentified genes linking insulin-like signaling to the regulation of GSC divisions [28], and their characterization may provide new insights into this cascade.

LKB1 was originally identified as an essential component of polarity establishment in the *C. elegans *zygote, and in the *Drosophila *oocyte and epithelium [82,83]. Furthermore, the artificial activation of LKB1 in mammalian intestinal epithelial cells upon overexpression of its cofactor STRAD induces their polarization and blocks their division in culture [84]. In fact, LKB1 was shown to phosphorylate and activate several AMPK-related kinases, including PAR-1/MARK and SAD/BRSK, both of which play important roles in polarity establishment during development [82,83,85,86]. It is however unclear whether LKB1 contributes to the regulation of GSC divisions by insulin-like signaling levels through its effects on cell polarity.

## Conclusion

In summary, evidence from *C. elegans *and *Drosophila *suggest that the general nutritional status of an organism, as reflected by the level of insulin-like signaling, regulates the production of a neuro-endocrine signal that is received by the GSCs and which dictates their division rate. This neuro-endocrine signal may target the insulin receptor itself (*Drosophila*), PTEN, and/or the LKB1-AMPK cascade (*C. elegans*) within the GSCs to link their proliferation rate with insulin-like signaling level, at least in part, in a FOXO-independent manner (*C. elegans*). Under the conditions where insulin-like signaling is low enough to completely block GSC divisions, the cells arrest at the G_2_/M checkpoint, although this has not been confirmed in *Drosophila*. These features may underlie the nutrient based regulation of cell divisions in several types of stem cell populations, and their better definition will be an important priority to understand the key mechanisms that control their proliferative capacity, both *in vivo *and *in vitro*, and whether cell or planar polarity is involved.

Whether the nutritional, insulin-dependent control of GSC proliferation rate somehow relies on microRNA-mediated control mechanisms is another important question that should be addressed. Although this model has been proposed [7], the two pathways may act in a completely parallel manner, as it seems to be the case regarding the interplay between the niche and the specification of GSC identity. Namely, no evidence involves microRNAs in insulin-like or nutrient-dependent regulation of GSC division rate. A possible reflection of this may however underlie the germline hyperplasia of *cki-1(RNAi) C. elegans *dauer larvae, since this microRNA pathway is believed to ultimately target a p21/p27 homolog.

Finally, GSCs share several features with most types of cancer cells, including sustained proliferation, the rate of which is sensitive to insulin signaling levels. Furthermore, several related cancer-predisposing syndromes result directly from germline mutations in central genes in the pathways that seem to couple GSC proliferation with organismal insulin signaling levels, including PTEN and LKB1. The identification of the downstream targets in this cascade will not only provide potential new candidates for cancer therapy, but may also uncover some rationale underlying the different characteristics of each of the cancer-predisposing syndromes associated with mutations in these genes.

In the light of this discussion, one interesting possibility that may account for some of the tumor suppressive effects of PTEN and LKB1 is their function in preventing division specifically when cells are more likely to acquire mutations, during cell cycle progression under nutrient depletion. Such mutant cells would therefore be predicted to have a higher mutational rate, and may therefore rapidly gain a stronger proliferative advantage, or other characteristics that affect key steps toward tumorigenesis.

## Competing interests

The author(s) declare that they have no competing interests.

## Authors' contributions

P.N. wrote the review, which was edited by R.R.
